# Full body illusion is associated with widespread skin temperature reduction

**DOI:** 10.3389/fnbeh.2013.00065

**Published:** 2013-07-25

**Authors:** Roy Salomon, Melanie Lim, Christian Pfeiffer, Roger Gassert, Olaf Blanke

**Affiliations:** ^1^Center for Neuroprosthetics, School of Life Sciences, Ecole Polytechnique Fédérale de LausanneLausanne, Switzerland; ^2^Laboratory of Cognitive Neuroscience, Brain Mind Institute, Ecole Polytechnique Fédérale de LausanneLausanne, Switzerland; ^3^Department of Health Sciences and Technology, Rehabilitation Engineering LaboratoryETH Zurich, Zurich, Switzerland; ^4^Department of Neurology, University HospitalGeneva, Switzerland

**Keywords:** bodily self-consciousness, body representation, body illusion, self-representation, body-ownership, neuroscience robotics

## Abstract

A central feature of our consciousness is the experience of the self as a unified entity residing in a physical body, termed bodily self-consciousness. This phenomenon includes aspects such as the sense of owning a body (also known as body ownership) and has been suggested to arise from the integration of sensory signals from the body. Several studies have shown that temporally synchronous tactile stimulation of the real body and visual stimulation of a fake or virtual body can induce changes in bodily self-consciousness, typically resulting in a sense of illusory ownership over the fake body. The present study assessed the effect of anatomical congruency of visuo-tactile stimulation on bodily self-consciousness. A virtual body was presented and temporally synchronous visuo-tactile stroking was applied simultaneously to the participants' body and to the virtual body. We manipulated the anatomical locations of the visuo-tactile stroking (i.e., on the back, on the leg), resulting in congruent stroking (stroking was felt and seen on the back or the leg) or incongruent stroking (i.e., stroking was felt on the leg and seen on the back). We measured self-identification with the virtual body and self-location as well as skin temperature. Illusory self-identification with the avatar as well as changes in self-location were experienced in the congruent stroking conditions. Participants showed a decrease in skin temperature across several body locations during congruent stimulation. These data establish that the full-body illusion (FBI) alters bodily self-consciousness and instigates widespread physiological changes in the participant's body.

## Introduction

Since William James's characterization of self-consciousness in the 19th century (James, 1890/1950), the psychological “self” has been the subject of much intrigue in the world of psychology, philosophy, and more recently, neuroscience. It has been proposed that the processing and integration of body-related information is important to develop a comprehensive neurobiological model of self-consciousness (Damasio, 2000; Jeannerod, 2006; Craig, 2009; Blanke, 2012). Recent advances in virtual reality (VR) technologies have enabled the investigation of bodily self-consciousness by providing subjects with ambiguous multisensory information about the location and appearance of their own body (Serino et al., 2008; Salomon et al., 2009, 2012). This has made it possible to study three important aspects of bodily self-consciousness and how they relate to the processing of bodily signals: self-identification with the body (the experience of owning a body), self-location (the experience of where I am in space), and first-person perspective (the experience of from where I perceive) (Blanke, 2012; Pfeiffer et al., 2013).

Although the sense of owning a body is often taken for granted, there are many cases of impaired sense of body ownership. For example, neurological patients with damage in the parietal lobe or insula will often neglect or deny ownership for body parts, one side of the body, or the entire body (e.g., Karnath et al., 2005; Vallar and Ronchi, 2009; Heydrich et al., 2010). Recent research has shown that hand ownership can also be manipulated in healthy individuals. Botvinick and Cohen's discovery of the rubber hand illusion (RHI) revealed that an illusory ownership for a rubber hand arises when synchronous visuo-tactile stimulation is administered to a person's occluded hand and that of a viewed rubber hand. This illusion is abolished when the tactile stimulation on the person's real hand is asynchronous to the stimulation seen on the rubber hand (Botvinick and Cohen, 1998) and can also be induced using VR technology (e.g., Slater et al., 2008; Evans and Blanke, 2013). As in other multisensory illusions, where specific combinations of multisensory information give rise to an erroneous subjective perception (e.g., McGurk and Macdonald, 1976), bodily illusions indicate states in which people report strong sensations of erroneous bodily self-consciousness due to an experimental manipulation of multisensory conflict between visual, proprioceptive, and tactile cues. The multisensory conflict between the visuo-tactile stimulation induces bodily illusions, reflecting alterations of bodily self-consciousness (Ehrsson, 2007; Blanke, 2012).

The RHI allows to investigate the integration of visual, tactile and proprioceptive signals and its importance for central body representation. However, more recent studies have shown that visuo-tactile mismatch may modify not only ownership of a body part, but may also induce ownership for a fake or virtual body (self-identification with a filmed or virtual body; Ehrsson, 2007; Petkova and Ehrsson, 2008), changes in self-location (Lenggenhager et al., 2007; Aspell et al., 2009), and changes in subjective first-person perspective (Ionta et al., 2011; Pfeiffer et al., 2013). The latter paradigm (i.e., full-body illusion, FBI) consists of participants viewing a video image on a head mounted display (HMD) that was linked to a video camera (placed 2 m behind the person) filming the participant's back from behind. Participants thus viewed the video image of their body while an experimenter stroked their back with a stick (the stroking was perceived by the participants on their back and also seen on the back of the virtual body). The HMD displayed the stroking of the virtual body either in real time or not (using an online video-delay or offline pre-recorded data), generating synchronous and asynchronous visuo-tactile stimulation.

The RHI and the FBI are most often quantified based on participants' responses to questionnaires and responses in different behavioral tasks. Several studies have shown that illusion conditions can elicit a change in the perceived location of the body or hand (Botvinick and Cohen, 1998; Tsakiris and Haggard, 2005; Lenggenhager et al., 2007), as well as changes in tactile processing (Pavani et al., 2000; Aspell et al., 2009; Zopf et al., 2010). Likewise, physiological measures have been employed and revealed heightened skin conductance responses (SCR) in reaction to threats to an illusorily embodied rubber hand (Armel and Ramachandran, 2003) or body (e.g., Ehrsson, 2007; Petkova and Ehrsson, 2008; Slater et al., 2008). The FBI has also been shown to affect pain perception by increasing pain thresholds (Hänsel et al., 2011). Additionally, differences in histamine reactivity (Barnsley et al., 2011) and the cooling of the participant's hand during the RHI (Moseley et al., 2008) have been found to accompany changes in illusory hand ownership. In summary, both the RHI and the FBI have been associated with physiological, behavioral, and tactile perceptual changes, whereas only the RHI has been linked to physiological changes in temperature and immune regulatory processes. These physiological changes have been suggested to reflect parasympathetic changes related to the modulations in body ownership. Such changes of temperature regulation can be found in several clinical conditions associated with disturbances in body ownership such as complex regional pain syndrome (Jänig and Baron, 2003; Moseley, 2005), anorexia (Lautenbacher et al., 1991), self-injurious behavior (Symons et al., 2001) as well as others (for more details of such clinical conditions see Table S1 in Moseley et al., 2008). It has been proposed that these modulations of temperature related to body ownership may be mediated though the insular cortex which has been shown to be involved in illusions of body ownership (e.g., Tsakiris, 2010; Blanke, 2012; Heydrich and Blanke, 2013) as well as coding of introspective information (Craig et al., 2000; Damasio et al., 2000; Craig, 2009).

While the FBI and the RHI are both induced by temporally synchronous visuo-tactile stroking of a seen body or hand with the participant's body or hand, the induced state of bodily self-consciousness differs in several aspects. For example, the RHI shows high sensitivity to both postural and anatomical congruence (Tsakiris and Haggard, 2005; Costantini and Haggard, 2007) as well as to the spatial distance between the seen and the touched hand. Thus, one study tested the effects of varying distance between the viewed rubber hand and the real hand and found that the illusion was strongest when the hands were near to each other and decayed rapidly when they were moved apart (Lloyd, 2007). Differently, FBIs can be induced when seeing a virtual body from a body-centered viewpoint (Petkova et al., 2011), but also from a distance (Ehrsson, 2007; Lenggenhager et al., 2007). Furthermore, classical FBI and RHI paradigms compare conditions in which a temporal conflict (and hence an anatomical conflict) exists (asynchronous visuo-tactile condition) to those with neither temporal nor anatomical conflicts (synchronous visuo-tactile conditions). However, to the best of our knowledge no experiment has compared the effects of an anatomical mismatch without any temporal conflict in the FBI.

Another difference between FBI and RHI relates to the anatomical specificity of the illusion. Studies have shown that during the RHI the proprioceptive drift may be constrained to the specific being finger stroked (Tsakiris and Haggard, 2005). However, the FBI, in which participants are typically stroked on their back, induces a change in self-location relating to the full body and its position in space rather than only the stroked region (Lenggenhager et al., 2007; Ionta et al., 2011). Recent findings from skin temperature measurements in the RHI have also shown a certain degree of specificity with decreased temperature in the illusion condition only in the stroked hand, but not for the contralateral hand nor for other non-stroked control sites [i.e., the ipsilateral ankle; (Moseley et al., 2008)]. This provides additional, physiological evidence that the RHI illusion induces a local and anatomically specific change in embodiment. The FBI, however, may induce a more widespread change in the body representation (at the trunk and potentially beyond), although no direct physiological evidence of such widespread changes has been provided.

Here, a novel robotic device for tactile stimulation (Duenas et al., 2011) is combined with VR to perform precise and reproducible visuo-tactile manipulations of the FBI. The robotic device was capable of independently stroking both the back (as in previous FBI studies) but also the legs, allowing us to explore if the FBI can be evoked by stroking of the legs. We investigated the following questions: Is anatomically congruent visuo-tactile stimulation necessary for the induction of the FBI (as has been shown previously for the RHI, but not yet tested for the FBI)? Would the induction of the FBI be accompanied with skin temperature reduction and will the temperature reduction be widespread or locally confined to the location of congruent visuo-tactile stroking as has been shown for the RHI (Moseley et al., 2008)? Participants were stroked by the robotic device on their back or leg while seeing anatomically congruent or incongruent visual feedback. Skin temperature, self-identification, self-location and tactile perception were measured. We hypothesized that (1) self-identification will be stronger during spatially-congruent versus incongruent conditions, and that (2) these illusion-inducing conditions will be associated with a reduction of skin temperature, which unlike in the RHI (Moseley et al., 2008) will not be specific to the location of visual tactile stimulation but rather widespread throughout all four recording sites. Following the results of Moseley et al. (2008) we expected that (3) response times (RTs) in the speeded tactile reaction would be longer in the congruent stroking conditions. Finally in line with previous experiments (e.g., Lenggenhager et al., 2009) we expected (4) a modulation of the RTs in the mental ball drop task as a function of visual-tactile stroking congruency.

## Methods

### Participants

Twenty-two volunteers (14 male, 8 female, mean age = 22.1 years, *SD* = 2.3) participated in the study. Participants had normal or corrected to normal vision and gave written informed consent. All participants were right handed. The study was performed in accordance to the ethical standards of the Declaration of Helsinki. The experimental protocol was approved by the local ethics committee: La Commission d'ethique de la recherche Clinique de la Faculte de Biologie et de Medecine—at the University of Lausanne, Switzerland.

### Materials and stimuli

**Figure 1:**
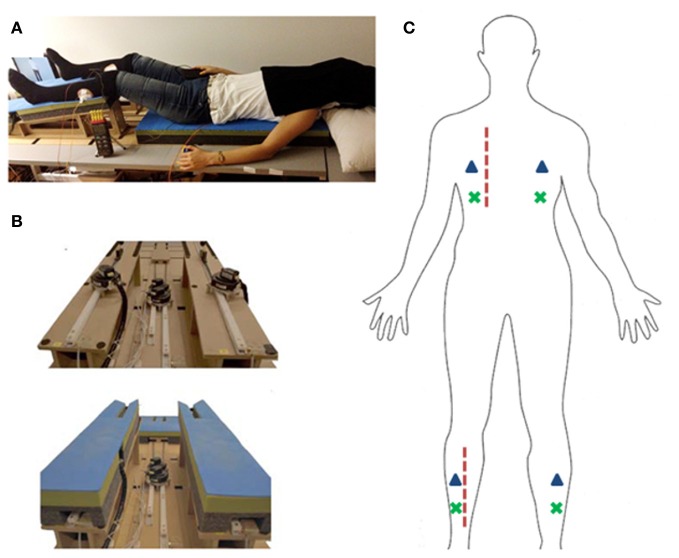
**Experimental setup. (A)** Picture of experimental setup. Participant lying on robotic stroking device. **(B)** Robotic stoking device shown from feet perspective. Top: padding removed for motor view. Bottom: robot with padding as used in the experiment. **(C)** Schematic representation of stroking regions (dashed red line), tactile vibrators (blue triangles), and thermocouple placement (green x).

We used a robotic stroking device, which allows for the precise application of tactile stimuli to ensure reproducibility and consistency across the experimental conditions and participants. The device used in this study is detailed in Duenas et al. (2011). In brief, the stroking mechanism consists of four individual stimulation modules—two at the back and two at the legs—driven by four ultrasonic motors (Shinsei Corp., Japan) over a rack and pinion gear. The stimulation modules move a polymer sphere that contacts the body, held by a polymer spring blade which ensures constant contact pressure (Figure 1C). Stroking movement is position controlled with a sampling rate of 200 Hz and can be controlled at velocities of 2–12 cm/s. The four stroking modules followed a sawtooth trajectory with a range of 20 cm for back-stroking modules and 16 cm for leg-stroking modules (left and right) at 0.4 Hz. LabVIEW software (National Instruments Corporation, version 2010b, www.ni.com/labview) was used to control the robotic stroking device.

EXpyVR (Custom in house software, http://lnco.epfl.ch/expyvr) was used for the programming and executing of the experiment, and recording of RTs. Responses were collected using a keypad (Targus Numeric Keypad AKP10US, www.targus.com).

Participants wore a V-Real Viewer 3D SVGA HMD (800 × 600 resolution, 35° field of view, www.vrealities.com/virtualviewer3d.html). On the HMD, participants viewed the virtual avatar from behind, with an overlay of the position and range of stroking corresponding to that of the robotic stroking modules (Duenas et al., 2011; Ionta et al., 2011).

The participants were outfitted with four tactile vibrators at the left back, right back, left leg, right leg. Each vibrator consisted of a small vibrating motor (Precision MicroDrives shaftless vibration motors, model 312).

Participants' skin temperature was measured with a HH309A Data Logger Thermometer (Omega, Stamford, USA) with four type K thermocouples and a real-time clock. The skin temperature of participants were measured at four locations, 4 cm below the tactile vibrators on the left back, right back, left leg, and right leg. The temperature at each location was recorded every 2 s over the entire course of each experimental block, with the start of the experiment coinciding with the beginning of the temperature recording. We tested the variance of the thermometer in seven participants using the same locations and durations as used in the experiment. The participants were lying in a relaxed supine position without and visual or tactile stimulation. The mean variance of skin temperature change was 0.0005°C.

### Experimental design

We used full-factorial design with a 2 (Visual Stroking: back, leg) ×2 (Tactile Stroking: back, leg) within-participant manipulation.

### Procedure

At the start of the experiment, participants were briefed about the stimuli they were going to experience and observe, and instructed on the different tasks they were to perform. All participants wore a white t-shirt and black socks which allowed for the placement of tactile vibrators and thermocouples on the skin. Participants were placed in a supine position on the robotic stroking device and wore a HMD covered by a black cloth to occlude peripheral vision. Participants held a ball (12 cm diameter) in their left hand and a response button device in their right hand.

A training session was carried out before the experiment, where participants saw a moving red dot against a black background and experienced the stroking first on their left back (15 s). This was followed by an auditory cue for a Mental Ball Dropping (MBD) Task (adapted from Lenggenhager et al., 2009; Ionta et al., 2011). The MBD task was used to measure self-location, by measuring the modulation of the experimental conditions on the judged time for an imaginary ball dropping from the participants' hand to reach the floor. First, participants pressed a button to indicate that they imagined releasing the ball from their hand (which was positioned close to the body at level with participants lying on the back). Secondly, participants held the button depressed during imagined ball dropping, and released the button to indicate that they imagined the ball hitting the floor. Button press duration (i.e., RT) were shown to be a sensitive measure of participants self-location in previous work (Lenggenhager et al., 2009).

Participants then performed a Speeded Tactile Reaction task. Tactile processing at different locations of the body was measured by a novel approach modified from the Temporal Order Judgments (TOJ) task, which showed that the RHI slowed tactile processing (Moseley et al., 2008). For the task in this experiment, participants' reaction times to vibration stimuli on four locations (left and right back and left and right leg) were tested. Each tactile vibrator was placed 5 cm laterally from the midpoint of the robot's stroking range. Participants felt four tactile vibrations (100 ms duration) at four different locations in random order during each trial, with random inter-stimulus intervals (0.5, 1, and 1.5 s) between vibrations. Participants were told to respond immediately each time they felt a vibration. Their RTs at the different locations were compared to a baseline measured in 40 practice trials (10 times at each location) before the actual experiment to correct for differences in tactile processing at different regions of the body. This procedure was repeated twice during the training session, with participants experiencing the stroking on their left leg during the second trial. Participants indicated their understanding of the tasks after the training session before proceeding on to the main experiment.

**Figure 2:**
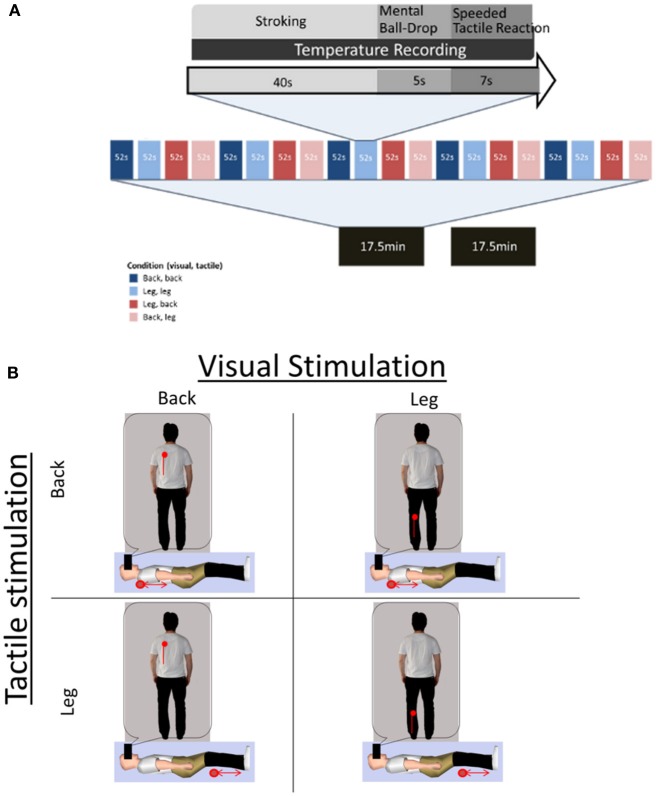
**Experimental paradigm and factorial design. (A)** Experimental procedure of each trial (top), each block (20 trials-middle), and full experiment (two blocks-bottom). **(B)** Factorial design of the experiment: The four panels show different visuo-tactile stroking conditions. Participants lay supine and received tactile stroking on the back or leg (red dot and arrow represent tactile stroking range). Participants observed a virtual body (vertical body) and viewed visual stroking on the back or the leg as a movement of a red dot (vertical line marks the extent of visual stimulation and was not presented to participants). Note that the viewed virtual body was aligned in the same plane as the participants' body and is rotated for presentation purposes only.

In each trial, participants were subjected to 40 s of stroking by the robotic device and observed a synchronous stroking pattern on a virtual body through the HMD. The stroking was applied on either their left back or left leg in each trial and participants were presented a synchronous movement of a red dot on either the left back or left leg of the virtual body (Figure 1). They were immediately prompted by an audio cue for a mental ball-drop and given 5 s to respond, followed by a Speeded Tactile Reaction task lasting 7 s. Each of the four experimental conditions (observed congruent back and leg stroking, observed incongruent back, and leg stroking) was presented five times in a single block, over two blocks (Figure 2).

The four conditions presented were spatially congruent back-stroking, spatially congruent leg-stroking, spatially incongruent back-stroking and spatially incongruent leg-stroking. The order of conditions was randomized for every block.

**Figure 3:**
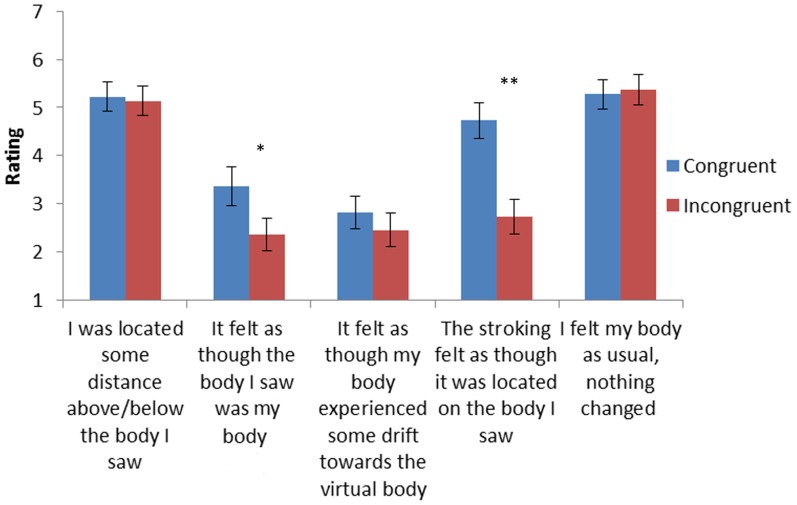
**Questionnaire ratings for Q1-5 (mean ± standard error).** *N* = 22. ^*^Indicates *p* < 0.002, and ^**^indicates *p* < 0.0001.

After having completed the experiment on the robotic device participants completed a questionnaire at the end of the experiment on a visual analogue scale (VAS) with scores ranging from −3 (absolutely NOT applicable) to +3 (absolutely applicable) pertaining to the two conditions, congruent, and incongruent stroking. All participants were aware of the difference between the two conditions. Questions were modified from (Lenggenhager et al., 2009; Ionta et al., 2011) and inquired about illusory touch, self-identification, and also contained control items (Figure 3).

Finally, participants were asked to give free verbal responses related to their sensations during the experiment. For each response they gave they were then prompted by the experimenter to reveal if this sensation was different for the congruent and incongruent conditions and if it differed for the locations of visuo-tactile stroking during the congruent condition (i.e., back-back vs. leg-leg). Responses were written down by the experimenter.

### Analysis

Self-identification questionnaire ratings were analyzed with 2 × 2 repeated measures ANOVA with Congruence (congruent/incongruent) and Question (Q1–5) as factors. Fisher's Least Significant Difference (LSD) analysis with threshold at *p* < 0.05 was used for all *post-hoc* comparisons. For presentation purposes the scale was then transformed into positive units resulting in a scale of 1–7 corresponding to the −3 (absolutely NOT applicable) to +3 (absolutely applicable) originally measured.

Processing of RTs from the MBD task included removing dataset outlier values exceeding two standard deviations from the participant's mean for each individual. Condition average RTs for four experimental conditions were calculated and statistically analyzed with a 2 × 2 repeated measures ANOVA with factors Visual stimulation location (back/leg) and Stroking location (back/leg).

Processing of RTs for the Speeded Tactile Reaction Task consisted of removing for each individual dataset outlier values exceeding two standard deviations from the participant's mean. These were analyzed with 2 × 2 × 4 repeated measures ANOVA with factors tactile stimulation location (left and right back, left and right leg), Visual stimulation location (back/leg), Stroking location (back/leg).

Temperature data during the 40 s (20 time points) of stroking were analyzed by means of independent repeated measures 2 × 2 × 4 ANOVA for each time point (1-20), with Visual stimulation location (back/leg), Stroking location (back/leg), and Thermometer location (left and right back, left and right leg) as factors. Changes in temperature (ΔT) were calculated by subtracting the skin temperature at the start of stroking (time point 1) from all subsequent time points (time point 2–20) for each trial. Bonferroni correction for multiple comparisons was used for statistical analysis of temperature.

Overall changes in temperature were analyzed using a repeated measures 2 × 2 ANOVA with Visual stimulation location (back/leg) and Stroking location (back/leg) as factors. This was done once for the time period corresponding to the onset of the illusion as well as for the overall experimental epoch.

For the analysis of temperature data, five participants were excluded from the analysis due to movements during the experiment which displaced the thermocouples. For the MBD task, outliers were discarded (total loss, 1.4% of trials, out of which 1 participant, whose mean reaction times were over 3 s, was excluded from analysis). For the analysis of RTs for the Speeded Tactile Reaction Task Trials outliers were removed (total loss, <1% of trials).

## Results

### Self-identification

Subjective responses are shown in Figure 3 and revealed stronger self-identification with the virtual body for congruent versus incongruent conditions [Q2: It felt as though the body I saw was as if it were my body; congruent trials *M* = 3.36, *SE* = 0.39; than incongruent trials: *M* = 2.36, *SE* = 0.33; *F*_(1, 21)_ = 7.21, *p* = 0.013]. There was also a significant congruence effect for Q4 (The stroking felt as though it was located on the body I saw) [*F*_(1, 21)_ = 34.22, *p* < 0.001], indicating illusory touch for the viewed body for the congruent condition (*M* = 4.7, *SE* = 0.36) that was larger than in the case of the incongruent condition (*M* = 2.7, *SE* = 0.35). The questions related to changes in self-location (Q1 and Q3) as well as the control question (Q5) showed no differences between the conditions.

### Self-location

Statistical analysis of RTs from the MBD task showed a trend for an interaction between visual and tactile input locations on participants' perception of self-location [*F*_(1, 20)_ = 3.65, *p* = 0.07]. *Post-hoc* comparisons revealed that participants had shorter RTs during the congruent condition (*M* = 872 ms, *SE* = 5 ms) than in the incongruent condition (*M* = 884 ms, *SE* = 5 ms, *p* = 0.03). When examining all visual and tactile stroking locations the back-congruent condition (*M* = 860 ms, *SE* = 5 ms) showed faster reaction times as compared to the incongruent leg-stroking condition (*M* = 890 ms, *SE* = 5 ms) (*p* < 0.01). This result indicates a change in self-location between congruent and incongruent conditions. No other effect was found for visual and tactile interaction (all *p* > 0.2).

### Temperature changes

No significant differences in temperature were found for the different measurement locations (left/right leg, left/right back *p* = 0.8 n.s.). There were no significant interactions between temperature measurement location and the other factors (all *p* > 0.25) at any of the 20 time points. This indicated that the cooling effect (see below) was not linked to any specific location of measurement. Hence, the measurements from all four locations were averaged.

**Figure 4:**
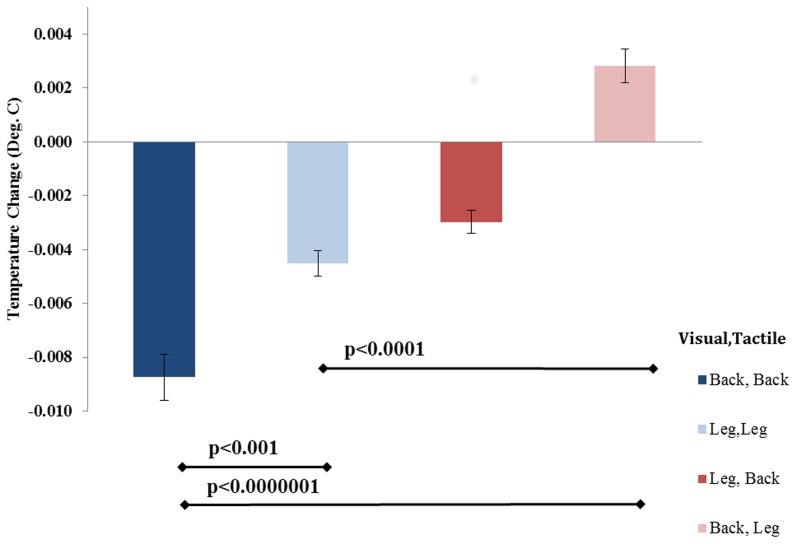
**Temperature data in all conditions.** Temperature changes across four locations and across all time points (mean ± standard error). *N* = 17.

Analysis of the temperature changes showed a strong effect of visual-tactile congruency on skin temperature [*F*_(1, 18)_ = 70.115, *p* < 0.001] with lower skin temperature in the congruent condition (*M* = −0.0066, *SE* = 0.00064) than in the incongruent condition (*M* = −0.00007, *SE* = 0.0002). The effect of location of stroking showed a trend [*F*_(1, 18)_ = 3.7881, *p* = 0.06] with lower temperature when the back was stroked (*M* = −0.0037, *SE* = 0.0004) than when the leg was stroked (*M* = −0.0029, *SE* = 0.0002). Finally the interaction between congruency and stroking location was also significant [*F*_(1, 18)_ = 55.268, *p* < 0.001]. *Post-hoc* Bonferroni tests indicated that this interaction was driven primarily by a large temperature difference between the back congruent (back-back) and back incongruent (back-leg) conditions (see Figure 4).

**Figure 5:**
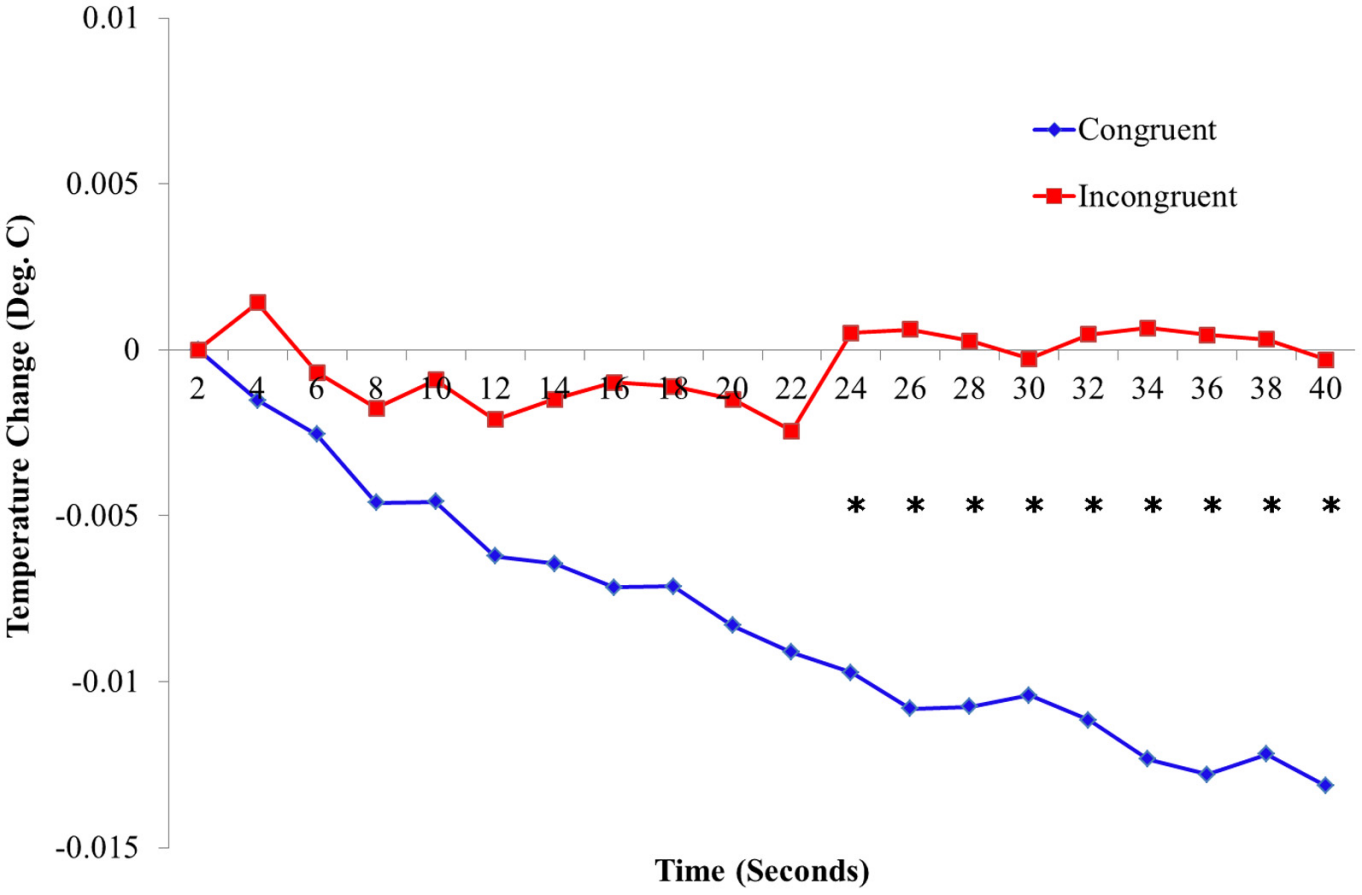
**Temperature time course data.** Temperature changes across four locations during 40 s of stroking for all conditions. Blue and Red lines represent temperature changes between congruent and both incongruent conditions, respectively. ^*^indicate locations of significant differences between congruent and incongruent conditions (individual ANOVA, *p* < 0.05, Bonferoni corrected). *N* = 17.

We then analyzed the evolution of the temperature changes over time. A separate ANOVA was conducted for each of the measured time points of the temperature data (see methods), hence removing any autocorrelations between timepoints. This revealed a significant effect of the interaction for visual and tactile stimulation location (e.g., back-back) starting from the 12th time point to the 20th time point (24–40 s of stroking) on the temperature [*F*_(1, 16)_ = 4.89, *p* = 0.042] (Figure 5 and Table S2A, for individual statistics at each time point). *Post-hoc* tests indicated that this interaction was driven by a significant reduction of skin temperature in congruent versus incongruent conditions for all measurement locations [*F*_(1, 16)_ = 70.11, *p* < 0.001]. We performed a permutation test on a binary vector containing 12 zeros and 8 ones (see Methods) to determine the probability of obtaining 8 consecutive significant time points within a 20 time point set. Within the simulated distribution of 1,000,000 permutations, only 94 occurrences of 8 consecutive time points occurred by chance, suggesting a *p*-value of 0.000094. There was no main effect of visual stimulation location, nor tactile stimulation location or skin temperature measurement location on changes in temperature throughout the 20 time points [*F*_(1, 16)_ = 0.26, *p* = 0.84] (see Table S2B, for individual statistics at each time point).

As the time course analysis indicated that the temperature change evolved over time we repeated the analysis for the overall temperature change and restricted it to the later epoch (24–40 s) of the trial. The results for the temperature change during this epoch showed larger differences and can be viewed in Figure S1.

### Speeded tactile reaction task

Results from the speeded tactile reaction task revealed no effects or interactions for visual stimulation, stroking, and location of vibration stimuli (left and right back, left and right leg) (all *p* > 0.1).

### Free responses

Free responses from subjects who reported bodily sensations (12/22) were collected and revealed that the congruent back stimulation was more pleasant than the congruent leg stimulation.

## Discussion

The present data revealed a continuous widespread decrease in skin temperature during the time course of the FBI. We found that the temperature decrease was not specific to the site of measurement, showing similar effects in all locations despite the considerable distance between the measurement locations. Furthermore, the present results show that anatomically congruent and temporally synchronous stimulation of the legs can induce a FBI. Additionally, we show that temporal synchrony between the visual and the tactile cue is not sufficient for inducing the FBI but an anatomical congruence of the visual and tactile stimulations is also required.

Subjective responses recorded via questionnaires and free responses are in accord with previous works using temporal synchrony manipulation (Lenggenhager et al., 2009; Ionta et al., 2011). These revealed that illusory self-identification with the visual virtual body (Q2) and illusory touch of the visuo-tactile event (Q4) were stronger in the congruent conditions. In their free responses most participants reported a stronger effect of illusory self-identification during congruent back-stroking trials as compared to congruent leg-stroking trials. Some responses include: “Back stroking led to a more effective way of thinking of the virtual body as a real body” and “Back-congruent condition was more pleasant and the feeling of my body being the virtual body was stronger for the back stroking than for leg stroking.”

Previous findings of participant's subjective feeling of ownership for a rubber hand or virtual body (as determined from responses to questionnaires) have shown differences between synchronous and asynchronous visual-tactile stimulations. These typically show stronger ownership for the fake body or body part in temporally synchronous conditions than temporally asynchronous conditions. However, it has been argued that these effects may be driven by a strong sensation of disownership for the body in the asynchronous condition. For instance, a study with 131 participants showed an ownership rating at +0.4 for synchronous conditions, but a larger negative magnitude of −1.2 for asynchronous conditions (Longo et al., 2008). As such, de Vignemont (2010), posits that the sense of ownership experienced by participants during synchronous stroking could possibly reflect participants' confidence in their judgment instead, with participants being more certain that the rubber hand did not belong to them in the asynchronous condition. The current study carefully controlled the synchrony of stroking—by means of a robotic system combined with VR—ensuring visual and tactile synchrony throughout all conditions and across different distant stimulation sites; hence the effects shown here are not related to a sense of disownership caused by temporally asynchronous stimulation. This extends the previous findings to suggest that anatomical congruence even in the absence of temporal conflicts is an important factor for illusory self-identification (as tested here), and potentially also for hand ownership as tested in the RHI.

Objective physiological measurements of participants' skin temperature indicated a cooling of the skin which was significant after 24 s of congruent visuo-tactile stimulation. Importantly, these changes (illusory self-identification, and cooling) were absent or weaker during incongruent stimulation. The present findings extend previous findings of cooling of skin temperature during illusory hand ownership (Moseley et al., 2008; Kammers et al., 2011). It has been suggested that the cooling of skin temperature during bodily illusions relates to a modulation of homeostatic activity due to a change in body representation. The alteration of the normal body representation by the illusion has been suggested to induce a “disownership” of the true body part, leading to a modulation of skin temperature, tactile processing (Moseley et al., 2008), pain thresholds (Hänsel et al., 2011), and even histamine reactivity (Barnsley et al., 2011). However, these previous studies have shown anatomically specific changes in line with the localized effects of the RHI. In the current study, and contrary to previous results the induction of the FBI resulted in a widespread temperature change in all four measured locations (legs and back). This is in accord with previous findings showing that despite localized visuo-tactile stimulation (on the back), the induced self-identification and self-location effects indicate changes encompassing the full body. The FBI has also demonstrated more flexible parameters than the RHI for self-identification with a false body. For instance, both male and female participants experience the same degree of illusion with the use of a male virtual avatar (Ionta et al., 2011) or male mannequin (Petkova and Ehrsson, 2008); furthermore the FBI can be induced for different viewpoints and different spatial distances between stroked and seen body (Ionta et al., 2011; Petkova et al., 2011). The RHI has been shown to be more restricted to specific spatial configurations (Costantini and Haggard, 2007; Lloyd, 2007). Our results build on these findings to suggest that the body representation is malleable enough to experience an illusion of the whole body, even when stroking is applied to the back or to the legs. While not measured in the current experiment, we speculate that this difference may be related to the difference in the size (or somatosensory receptor density) of the tactile receptive fields on the back/leg versus the hand (Gardner, 1988; Nakamura et al., 1998; Kurth et al., 2000). The larger receptive fields on the back and leg regions allow only a much coarser somatosensory resolution which may in turn lead to a less spatially specific and hence more global illusion resolution.

While not tested in this experiment, a possible mechanism for the effects of illusory self-identification on body temperature, brought about by visuo-tactile manipulation, could involve the insula, which is known to be one of the centers of activation during cross-modal visual and somatosensory activation (Bottini et al., 1995; Hadjikhani and Roland, 1998). The insula has also been found to be the main cortical substrate involved in discriminating innocuous thermal sensation (Craig et al., 2000), thermal regulation (Maihöfner et al., 2002) and is also found to play a critical role in the sense of limb ownership (Baier and Karnath, 2008; Karnath and Baier, 2010). It has thus been postulated to be the key neural substrate that mediates the influence of temperature on interoceptive processes (Kang et al., 2011).

It is noteworthy to mention that changes in body temperature found in the present study were highly significant, but very small (i.e., in the range of 0.006–0.014°C); those reported in the original experiment by Moseley [and as measured by a handheld thermometer device (Rayek)] were larger and around 0.24°C (Moseley et al., 2008 Exp. 1–3). There are several methodological differences between both experiments which may account for the discrepancies. First, the experiment by Moseley used the RHI as opposed to a FBI which may be associated with different temperature effects. In the previous RHI experiment the cooling effects were found for the hand which may have different thermal variation than the back and thigh that were measured in the present experiment. Also, in Moseley's experiment, temperature was recorded by means of a hand-held thermometer with readings every 30 s for 7–8 min, giving five readings for each location (stimulated hand, unstimulated hand, and ipsilateral ankle) in each trial. Here, we used a 4-channel thermometer with thermocouples as temperature sensors to record temperature every 2 s for 40 s, giving 20 readings for each location in each trial. The constant recording of temperature throughout the experiment allowed for the collection of a more continuous and well-controlled dataset, enabling a rigorous analysis of the temperature changes induced by the illusion. Finally, the shorter trial durations may also have affected the magnitude of the temperature change, as the trials in our experiment were considerably shorter than those used by Moseley et al. (40 s vs. 7–8 min). In Moseley et al. (2008) the temperature changes after 24 s (Figure 1B) are of a similar magnitude to those reported here, and the peak temperature change is found after about 4 min of stroking. Thus, the temperature change observed in our experiment may not have reached its asymptote in our shorter trails.

With a continuous temperature measurement at a high sampling rate, this study offers novel evidence, based on objective measurements, of the onset of physiological changes associated with illusory self-identification with a virtual body. Differences in skin temperature during the four conditions occurred from the start of stroking, with the gradual temperature change of the skin during both congruent trials, and minor fluctuations of temperature during incongruent trials. However, the differences among the conditions only became significant after 24 s of stroking. Previous research has shown that bodily illusions require time for their induction [i.e., ~11 s for the RHI (Ehrsson et al., 2004; Kammers et al., 2009)]. Therefore, we predicted that the temperature change would show similar dynamics and would require some time for induction. This is also in line with the findings of the Moseley et al. (2008) paper showing that the temperature change evolved over several minutes in the RHI (Moseley et al., 2008) (Figure 1B). These findings suggest that the psychological feeling of illusory self-identification during the FBI may be linked to the increasing magnitude of a widespread cooling of the body (at least with respect to those body parts sampled in the present study). We have argued earlier (Blanke, 2012) that differences in bodily self-consciousness between RHI and FBI might be related to the relevance of the stroked body regions for a global representation of the bodily self. Specifically, peripheral body regions might be less crucial than trunk regions for global aspects of the bodily self.

The measurement of self-location using the MBD task showed that participants had shorter RTs during congruent visuo-tactile stroking conditions. This result indicates a change in perceived self-location between the same conditions that were associated with changes in body temperature and self-identification with the virtual body. Neuroimaging experiments directly comparing visuo-tactile conflicts with and without temporal mismatch are necessary to investigate how changes in self-location map to changes in illusory self-identification and temperature change (Lenggenhager et al., 2009; Ionta et al., 2011).

## Conclusions

The findings of the current study have important implications for the understanding of bodily self-consciousness. They demonstrate for the first time that changes in full body self-consciousness induced by synchronous visuo-tactile stimulation relate to systematic and successive changes in skin temperature. Contrary to results from the RHI, these temperature changes are widespread and involve a cooling which spreads to both sides of the body as well as to regions anatomically distant from the stimulated region. These findings are compatible with the thesis that the FBI alters more global aspects of bodily self-consciousness as compared to the more local and limb-specific changes induced by the RHI (Blanke and Metzinger, 2009). This extends previous findings regarding differences in the behavioral and neural mechanisms of the FBI and RHI (Ehrsson et al., 2004; Ionta et al., 2011) suggesting that the sense of ownership over a limb and the full body may be quite different. Additionally, our results show that temporally synchronous stimulation between a seen body part and a different, touched body part is not sufficient for the induction of illusory self-identification with a virtual body and cooling of one's own body; temporally synchronous stimulation needs to be applied with an anatomical congruency. Our results highlight that the sense of ownership is a spatially multifaceted experience affecting both explicit as well as implicit bodily measures.

### Conflict of interest statement

The authors declare that the research was conducted in the absence of any commercial or financial relationships that could be construed as a potential conflict of interest.

## References

[B1] Armel K. C. Ramachandran V. S. (2003). Projecting sensations to external objects: evidence from skin conductance response. Proc. Biol. Sci. 270, 1499–1506 10.1098/rspb.2003.2364 12965016 PMC1691405

[B2] Aspell J. Lenggenhager B. Blanke O. (2009). Keeping in touch with one's self: multisensory mechanisms of self-consciousness. PLoS ONE 4:e6488 10.1371/journal.pone.0006488 19654862 PMC2715165

[B3] Baier B. Karnath H. O. (2008). Tight link between our sense of limb ownership and self-awareness of actions. Stroke 39, 486–488 10.1161/STROKEAHA.107.495606 18162622

[B4] Barnsley N. McAuley J. Mohan R. Dey A. Thomas P. Moseley G. (2011). The rubber hand illusion increases histamine reactivity in the real arm. Curr. Biol. 21, R945–R946 10.1016/j.cub.2011.10.039 22153159

[B5] Blanke O. (2012). Multisensory brain mechanisms of bodily self-consciousness. Nat. Rev. Neurosci. 13, 556–571 10.1038/nrn3292 22805909

[B6] Blanke O. Metzinger T. (2009). Full-body illusions and minimal phenomenal selfhood. Trends Cogn. Sci. 13, 7–13 10.1016/j.tics.2008.10.003 19058991

[B7] Bottini G. Paulesu E. Sterzi R. Warburton E. Wise R. Vallar G. (1995). Modulation of conscious experience by peripheral sensory stimuli. Nature 376, 778–781 10.1038/376778a0 7651537

[B8] Botvinick M. Cohen J. (1998). Rubber hands ‘feel’touch that eyes see. Nature 391, 756 10.1038/35784 9486643

[B9] Costantini M. Haggard P. (2007). The rubber hand illusion: sensitivity and reference frame for body ownership. Conscious. Cogn. 16, 229–240 10.1016/j.concog.2007.01.001 17317221

[B10] Craig A. (2009). How do you feel–now? The anterior insula and human awareness. Nat. Rev. Neurosci. 10, 59 10.1038/nrn2555 19096369

[B11] Craig A. D. Chen K. Bandy D. Reiman E. M. (2000). Thermosensory activation of insular cortex. Nat. Neurosci. 3, 184–190 10.1038/72131 10649575

[B12] Damasio A. (2000). The Feeling of What Happens: Body and Emotion in the Making of Consciousness, (Vintage: Harvest Books), 213–215

[B13] Damasio A. R. Grabowski T. J. Bechara A. Damasio H. Ponto L. L. Parvizi J. (2000). Subcortical and cortical brain activity during the feeling of self-generated emotions. Nat. Neurosci. 3, 1049–1056 10.1038/79871 11017179

[B14] De Vignemont F. (2010). Body schema and body image—Pros and cons. Neuropsychologia 48, 669–680 10.1016/j.neuropsychologia.2009.09.022 19786038

[B15] Duenas J. Chapuis D. Pfeiffer C. Martuzzi R. Ionta S. Blanke O. (2011). Neuroscience robotics to investigate multisensory integration and bodily awareness. Conf. Proc. IEEE Eng. Med. Biol. Soc. 8348–8352 10.1109/IEMBS.2011.6092059 22256283

[B16] Ehrsson H. Spence C. Passingham R. (2004). That's my hand! Activity in premotor cortex reflects feeling of ownership of a limb. Science 305, 875 10.1126/science.1097011 15232072

[B17] Ehrsson H. H. (2007). The experimental induction of out-of-body experiences. Science 317, 1048 10.1126/science.1142175 17717177

[B18] Evans N. Blanke O. (2013). Shared electrophysiology mechanisms of body ownership and motor imagery. Neuroimage 64, 216–228 10.1016/j.neuroimage.2012.09.027 22995776

[B19] Gardner E. P. (1988). Somatosensory cortical mechanisms of feature detection in tactile and kinesthetic discrimination. Can. J. Physiol. Pharmacol. 66, 439–454 10.1139/y88-074 3139269

[B20] Hadjikhani N. Roland P. E. (1998). Cross-modal transfer of information between the tactile and the visual representations in the human brain: a positron emission tomographic study. J. Neurosci. 18, 1072–1084 9437027 10.1523/JNEUROSCI.18-03-01072.1998 PMC6792755

[B21] Hänsel A. Lenggenhagerl B. Känell R. Curatolol M. Blankel O. (2011). Seeing and identifying with a virtual body decreases pain perception. Eur. J. Pain 15, 874–879 10.1016/j.ejpain.2011.03.013 21570328

[B22] Heydrich L. Blanke O. (2013). Distinct illusory own-body perceptions caused by damage to posterior insula and extrastriate cortex. Brain 136, 790–803 10.1093/brain/aws364 23423672

[B23] Heydrich L. Dieguez S. Grunwald T. Seeck M. Blanke O. (2010). Illusory own body perceptions: case reports and relevance for bodily self-consciousness. Conscious. Cogn. 19, 702–710 10.1016/j.concog.2010.04.010 20663690

[B24] Ionta S. Heydrich L. Lenggenhager B. Mouthon M. Fornari E. Chapuis D. (2011). Multisensory mechanisms in temporo-parietal cortex support self-location and first-person perspective. Neuron 70, 363–374 10.1016/j.neuron.2011.03.009 21521620

[B25] James W. (1890/1950). The Principles of Psychology. New York, NY: Dover Publications

[B26] Jänig W. Baron R. (2003). Complex regional pain syndrome: mystery explained? Lancet Neurol. 2, 687–697 10.1016/S1474-4422(03)00557-X 14572737

[B27] Jeannerod M. (2006). Motor Cognition: what Actions Tell the Self. Oxford: Oxford University Press

[B28] Kammers M. De Vignemont F. Verhagen L. Dijkerman H. C. (2009). The rubber hand illusion in action. Neuropsychologia 47, 204–211 10.1016/j.neuropsychologia.2008.07.028 18762203

[B29] Kammers M. P. M. Rose K. Haggard P. (2011). Feeling numb: temperature, but not thermal pain, modulates feeling of body ownership. Neuropsychologia 49, 1316–1321 10.1016/j.neuropsychologia.2011.02.039 21354190

[B30] Kang Y. Williams L. E. Clark M. S. Gray J. R. Bargh J. A. (2011). Physical temperature effects on trust behavior: the role of insula. Soc. Cogn. Affect. Neurosci. 6, 507–515 10.1093/scan/nsq077 20802090 PMC3150863

[B31] Karnath H. O. Baier B. (2010). Anosognosia for hemiparesis and hemiplegia: disturbed sense of agency and body ownership, in The Study of Anosognosia, ed Prigatano G. P. (Oxford: Oxford university Press), 39–62

[B32] Karnath H. O. Baier B. Nägele T. (2005). Awareness of the functioning of one's own limbs mediated by the insular cortex? J. Neurosci. 25, 7134–7138 10.1523/JNEUROSCI.1590-05.2005 16079395 PMC6725240

[B33] Kurth R. Villringer K. Curio G. Wolf K. J. Krause T. Repenthin J. (2000). fMRI shows multiple somatotopic digit representations in human primary somatosensory cortex. Neuroreport 11, 1487–1491 10.1097/00001756-200005150-00025 10841363

[B34] Lautenbacher S. Pauls A. M. Strian F. Pirke K.-M. Krieg J.-C. (1991). Pain sensitivity in anorexia nervosa and bulimia nervosa. Biol. Psychiatry 29, 1073–1078 10.1016/0006-3223(91)90249-L 1873371

[B35] Lenggenhager B. Mouthon M. Blanke O. (2009). Spatial aspects of bodily self-consciousness. Conscious. Cogn. 18, 110–117 10.1016/j.concog.2008.11.003 19109039

[B36] Lenggenhager B. Tadi T. Metzinger T. Blanke O. (2007). Video ergo sum: manipulating bodily self-consciousness. Science 317, 1096 10.1126/science.1143439 17717189

[B37] Lloyd D. M. (2007). Spatial limits on referred touch to an alien limb may reflect boundaries of visuo-tactile peripersonal space surrounding the hand. Brain Cogn. 64, 104–109 10.1016/j.bandc.2006.09.013 17118503

[B38] Longo M. R. Cardozo S. Haggard P. (2008). Visual enhancement of touch and the bodily self. Conscious. Cogn. 17, 1181–1191 10.1016/j.concog.2008.01.001 18294867

[B39] Maihöfner C. Kaltenhäuser M. Neundörfer B. Lang E. (2002). Temporo-spatial analysis of cortical activation by phasic innocuous and noxious cold stimuli–a magnetoencephalographic study. Pain 100, 281 10.1016/S0304-3959(02)00276-2 12467999

[B40] McGurk H. Macdonald J. (1976). Hearing lips and seeing voices. Nature 264, 746–748 1012311 10.1038/264746a0

[B41] Moseley G. L. (2005). Distorted body image in complex regional pain syndrome. Neurology 65, 773 10.1212/01.wnl.0000174515.07205.11 16157921

[B42] Moseley G. L. Olthof N. Venema A. Don S. Wijers M. Gallace A. (2008). Psychologically induced cooling of a specific body part caused by the illusory ownership of an artificial counterpart. Proc. Natl. Acad. Sci. U.S.A. 105, 13169–13173 10.1073/pnas.0803768105 18725630 PMC2529116

[B43] Nakamura A. Yamada T. Goto A. Kato T. Ito K. Abe Y. (1998). Somatosensory homunculus as drawn by MEG. Neuroimage 7, 377–386 10.1006/nimg.1998.0332 9626677

[B44] Pavani F. Spence C. Driver J. (2000). Visual capture of touch: out-of-the-body experiences with rubber gloves. Psychol. Sci. 11, 353 10.1111/1467-9280.00270 11228904

[B45] Petkova V. I. Ehrsson H. H. (2008). If I were you: perceptual illusion of body swapping. PLoS ONE 3:e3832 10.1371/journal.pone.0003832 19050755 PMC2585011

[B46] Petkova V. I. Khoshnevis M. Ehrsson H. H. (2011). The perspective matters! Multisensory integration in ego-centric reference frames determines full-body ownership. Front. Psychol. 2:35 10.3389/fpsyg.2011.00035 21687436 PMC3108400

[B47] Pfeiffer C. Lopez C. Schmutz V. Duenas J. A. Martuzzi R. Blanke O. (2013). Multisensory origin of the subjective first-person perspective: visual, tactile, and vestibular mechanisms. PLoS ONE 8:e61751 10.1371/journal.pone.0061751 23630611 PMC3632612

[B48] Salomon R. Malach R. Lamy D. (2009). Involvement of the intrinsic/default system in movement-related self recognition. PLoS ONE 4:e7527 10.1371/journal.pone.0007527 19844584 PMC2760765

[B49] Salomon R. Van Elk M. Aspell J. E. Blanke O. (2012). I feel who I see: visual body identity affects visual–tactile integration in peripersonal space. Conscious. Cogn. 21, 1355–1364 10.1016/j.concog.2012.06.012 22832215

[B50] Serino A. Pizzoferrato F. Ladavas E. (2008). Viewing a face (especially one's own face) being touched enhances tactile perception on the face. Psychol. Sci. 19, 434 10.1111/j.1467-9280.2008.02105.x 18466402

[B51] Slater M. Perez-Marcos D. Ehrsson H. H. Sanchez-Vives M. V. (2008). Towards a digital body: the virtual arm illusion. Front. Hum. Neurosci. 2:6 10.3389/neuro.09.006.2008 18958207 PMC2572198

[B52] Symons F. J. Sutton K. A. Bodfish J. W. (2001). Preliminary study of altered skin temperature at body sites associated with self-injurious behavior in adults who have developmental disabilities. Am. J. Ment. Retard. 106, 336–343 10.1352/0895-8017(2001)106<0336:PSOAST>2.0.CO;2 11414874

[B53] Tsakiris M. (2010). My body in the brain: a neurocognitive model of body-ownership. Neuropsychologia 48, 703–712 10.1016/j.neuropsychologia.2009.09.034 19819247

[B54] Tsakiris M. Haggard P. (2005). The rubber hand illusion revisited: visuotactile integration and self-attribution. J. Exp. Psychol. Hum. Percept. Perform. 31, 80 10.1037/0096-1523.31.1.80 15709864

[B55] Vallar G. Ronchi R. (2009). Somatoparaphrenia: a body delusion. A review of the neuropsychological literature. Exp. Brain Res. 192, 533–551 10.1007/s00221-008-1562-y 18813916

[B56] Zopf R. Savage G. Williams M. A. (2010). Crossmodal congruency measures of lateral distance effects on the rubber hand illusion. Neuropsychologia 48, 713–725 10.1016/j.neuropsychologia.2009.10.028 19913040

