# Supplementary material for: Full body illusion is associated with widespread skin temperature reduction
Source: Front Behav Neurosci. 2013 Jul 25;7:65. doi: 10.3389/fnbeh.2013.00065 (PMC3724056; doi:10.3389/fnbeh.2013.00065)
Supplement: Supplementary file 1 [file DataSheet1.DOCX]

**Supplementary Material: Full Body illusion is associated with widespread skin temperature reduction.** *Salomon et al. , 2013*

*Table1: Questions participants answered on a VAS, and their relevant evaluation of participant’s experience
* indicates questions in which participants’ responses differed significantly between congruent and incongruent conditions*

|  | Wording of Question | Evaluation |
| --- | --- | --- |
| *Q1* | I was located some distance above/below the body I saw | Experience of self-location |
| *Q2** | It felt as though the body I saw was as if it were my body | Identification with the visual virtual body |
| *Q3* | It felt as though my body experienced some drift towards the virtual body | Experience of drift in self-location |
| *Q4** | The stroking felt as though it was located on the body I saw | Experience of the visuo-tactile event |
| *Q5* | I felt my body as usual, nothing changed. | Control |

|  | | | | | |
| --- | --- | --- | --- | --- | --- |
| visual stimulation location * stroking location | | | | | |
| sample | Type III Sum of Squares | df | Mean Square | F | Sig. |
| 1.00 | .000 | 1 | .000 | . | . |
| 2.00 | .000 | 1 | .000 | 11.789 | .003 |
| 3.00 | .000 | 1 | .000 | 1.097 | .311 |
| 4.00 | .000 | 1 | .000 | 1.723 | .208 |
| 5.00 | .000 | 1 | .000 | 2.026 | .174 |
| 6.00 | .001 | 1 | .001 | 2.558 | .129 |
| 7.00 | .001 | 1 | .001 | 5.643 | .030 |
| 8.00 | .002 | 1 | .002 | 4.531 | .049 |
| 9.00 | .002 | 1 | .002 | 4.577 | .048 |
| 10.00 | .002 | 1 | .002 | 3.398 | .084 |
| 11.00 | .002 | 1 | .002 | 3.190 | .093 |
| 12.00 | .005 | 1 | .005 | 7.831 | *.013 |
| 13.00 | .007 | 1 | .007 | 8.033 | *.012 |
| 14.00 | .006 | 1 | .006 | 7.859 | *.013 |
| 15.00 | .005 | 1 | .005 | 5.697 | *.030 |
| 16.00 | .007 | 1 | .007 | 6.436 | *.022 |
| 17.00 | .008 | 1 | .008 | 6.575 | *.021 |
| 18.00 | .008 | 1 | .008 | 6.269 | *.023 |
| 19.00 | .007 | 1 | .007 | 4.845 | *.043 |
| 20.00 | .007 | 1 | .007 | 4.885 | *.042 |

*Table 2a. Statistical output for visual and tactile interaction across 20 time points*

|  | | | | | |
| --- | --- | --- | --- | --- | --- |
| visual stimulation location * stroking location * temperature measurement location | | | | | |
| sample | Type III Sum of Squares | df | Mean Square | F | Sig. |
| 1.00 | .000 | 3 | .000 | . | . |
| 2.00 | .000 | 3 | 9.547E-005 | 1.043 | .382 |
| 3.00 | .000 | 3 | 4.449E-005 | .268 | .848 |
| 4.00 | .001 | 3 | .000 | .554 | .648 |
| 5.00 | .001 | 3 | .000 | .734 | .537 |
| 6.00 | .001 | 3 | .000 | .863 | .467 |
| 7.00 | .001 | 3 | .000 | 1.107 | .355 |
| 8.00 | .001 | 3 | .000 | .683 | .567 |
| 9.00 | .001 | 3 | .000 | .439 | .726 |
| 10.00 | .001 | 3 | .000 | .680 | .569 |
| 11.00 | .001 | 3 | .000 | .562 | .642 |
| 12.00 | .002 | 3 | .001 | .676 | .571 |
| 13.00 | .002 | 3 | .001 | .704 | .554 |
| 14.00 | .002 | 3 | .001 | .627 | .601 |
| 15.00 | .002 | 3 | .001 | .499 | .684 |
| 16.00 | .003 | 3 | .001 | .601 | .617 |
| 17.00 | .003 | 3 | .001 | .707 | .553 |
| 18.00 | .005 | 3 | .002 | 1.034 | .386 |
| 19.00 | .007 | 3 | .002 | 1.327 | .277 |
| 20.00 | .007 | 3 | .002 | 1.298 | .286 |

*Table 2b. Statistical output for visual, tactile and measurement location interaction across 20 time points of temperature recording.*


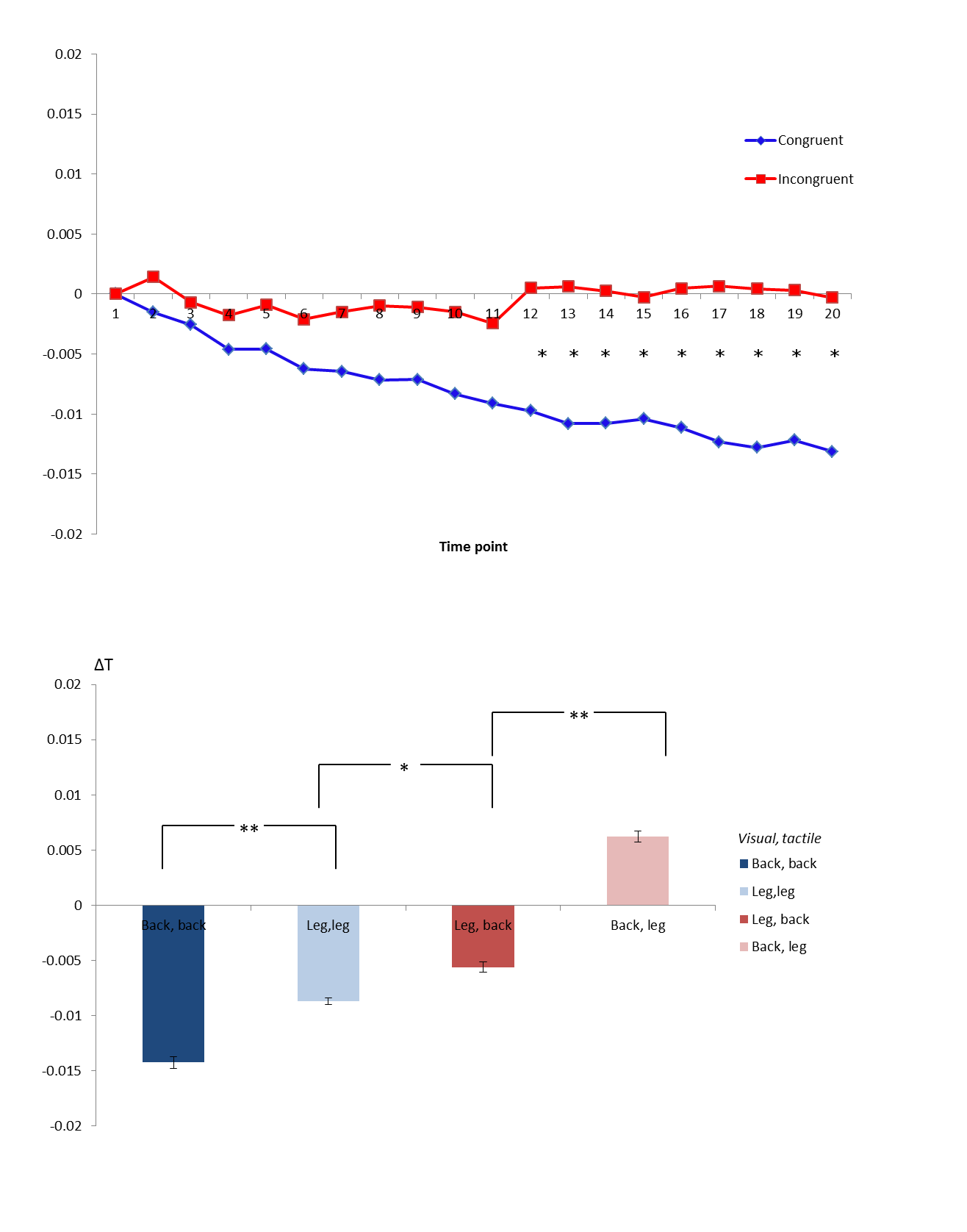


Supplementary Figure 1. Temperature changes across all locations during epoch of interest (24-40s), (mean ± standard error).
